# Supplementary material for: Transcriptomic Profiling of Buds Unveils Insights into Floral Initiation in Tea-Oil Tree (Camellia oleifera ‘changlin53’)
Source: Plants (Basel). 2025 Jul 30;14(15):2348. doi: 10.3390/plants14152348 (PMC12349429; doi:10.3390/plants14152348)
Supplement: Supplementary file 1 [file plants-14-02348-s001.zip › Supplementary figures.pptx]

## Slide 1
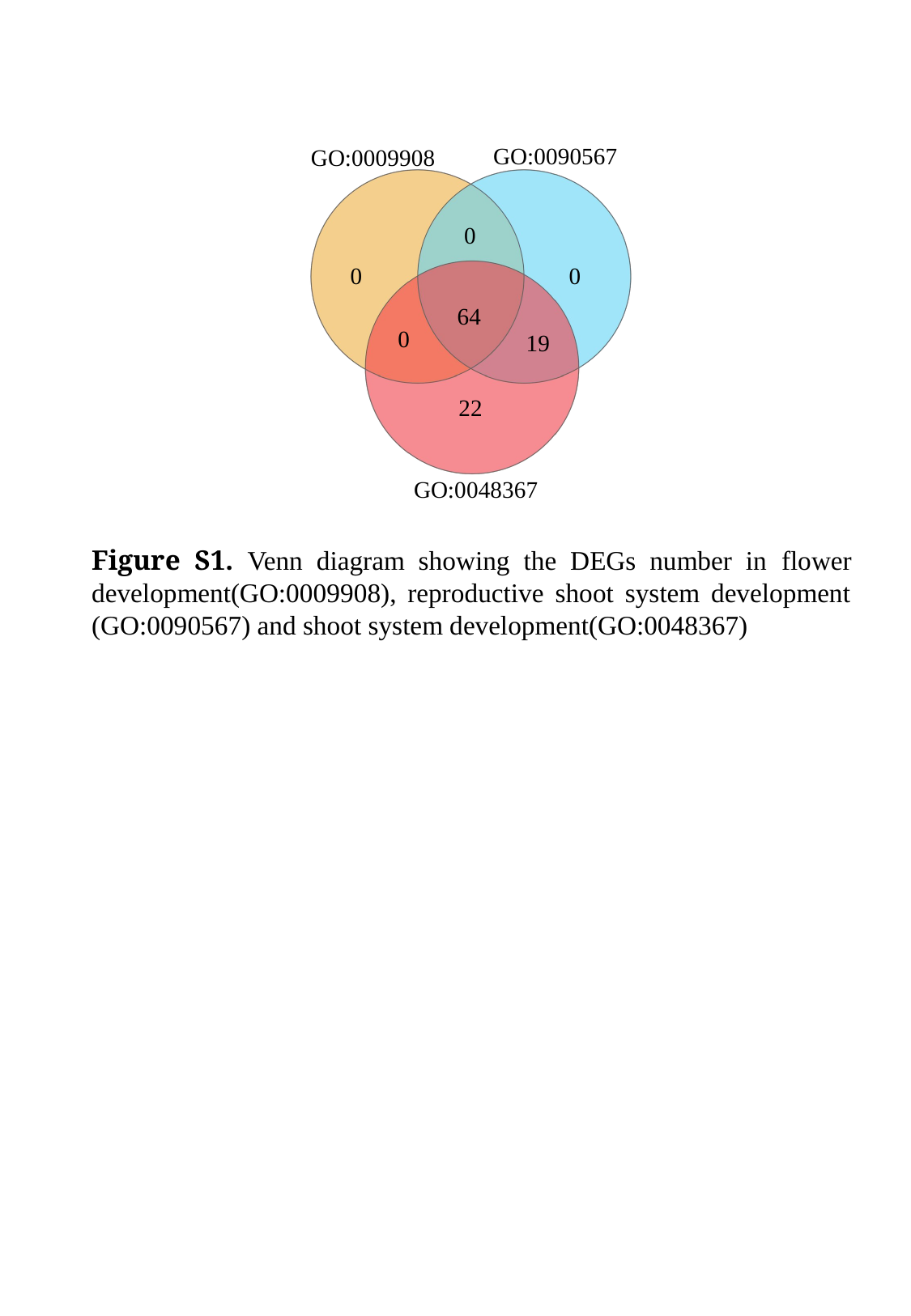

GO:0090567
GO:0009908
0
0
0
64
0
19
22
GO:0048367
Figure S1. Venn diagram showing the DEGs number in flower development(GO:0009908), reproductive shoot system development (GO:0090567) and shoot system development(GO:0048367)

## Slide 2
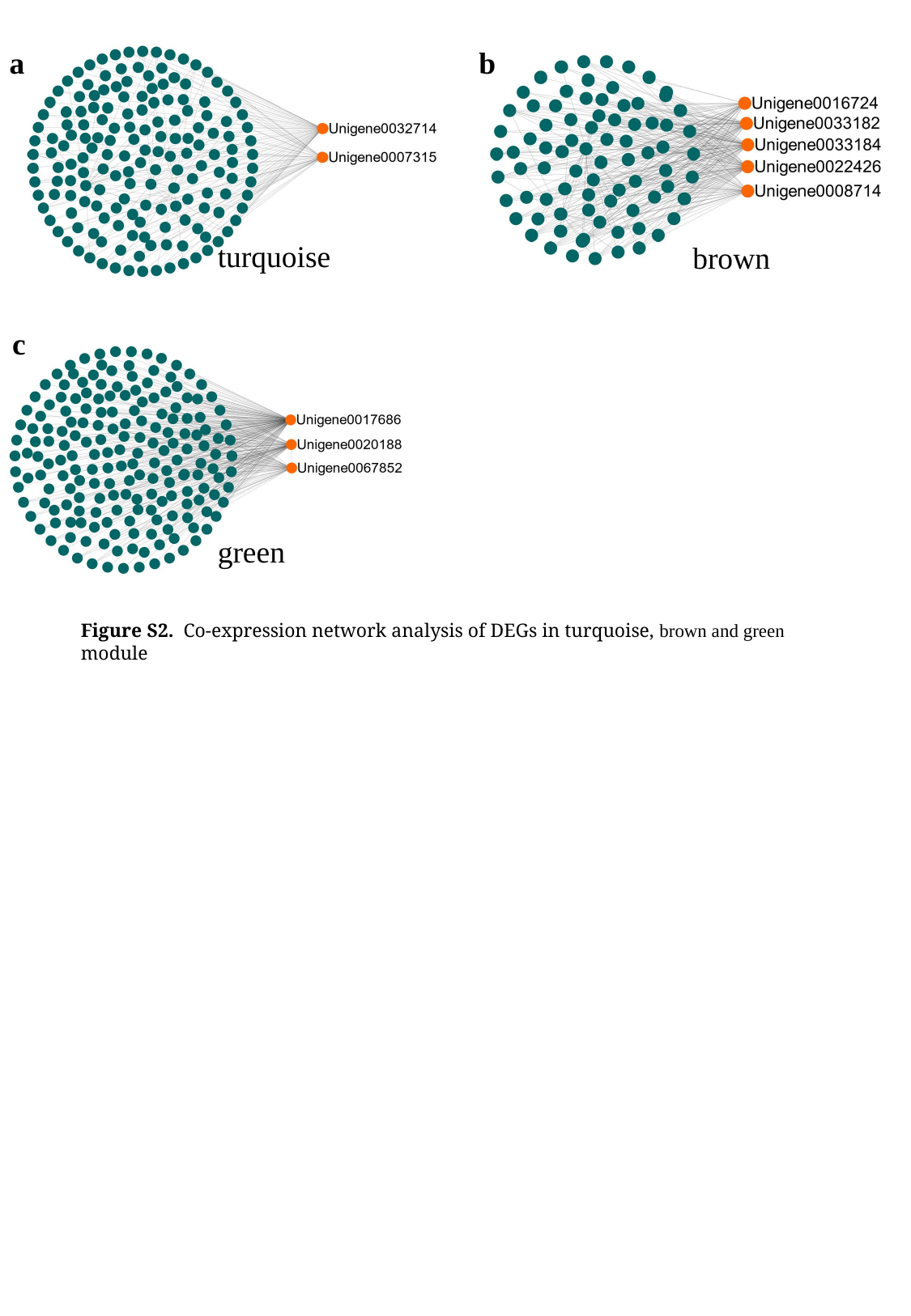

a
b
turquoise
brown
green
c
Figure S2. Co-expression network analysis of DEGs in turquoise, brown and green module

## Slide 3
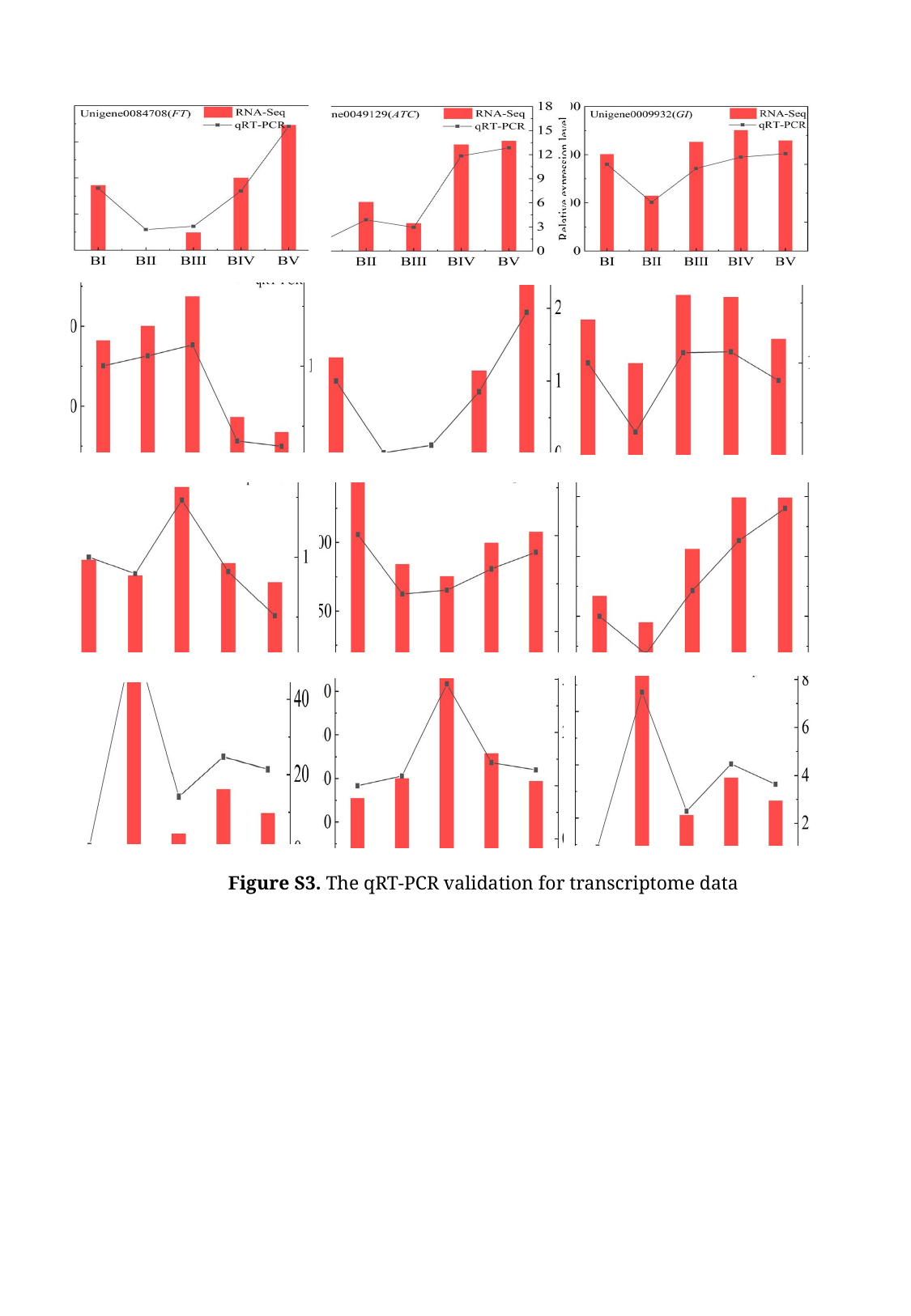

Figure S3. The qRT-PCR validation for transcriptome data

## Slide 4
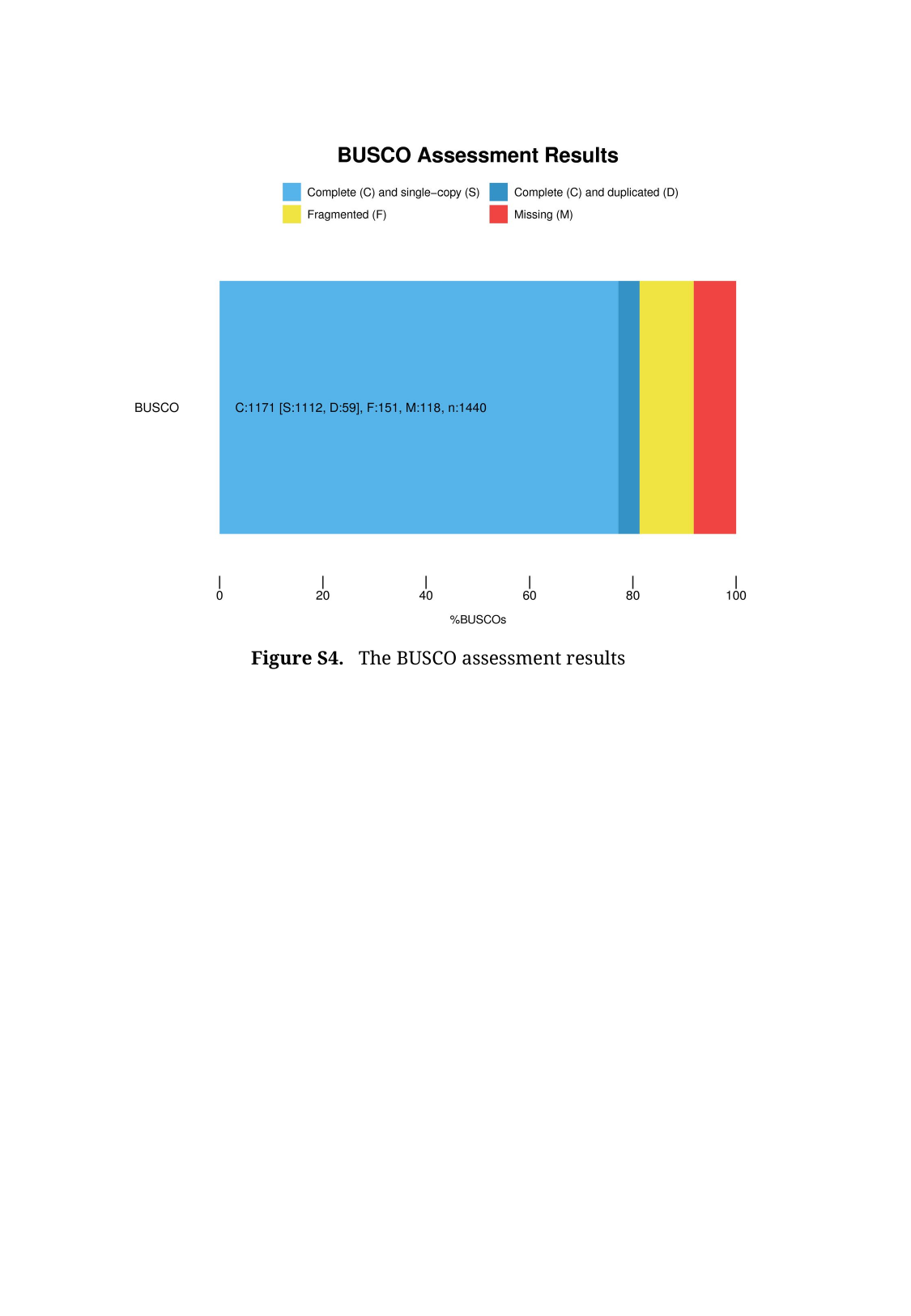

Figure S4. The BUSCO assessment results
